# Adjuvant Antitumor Immunity Contributes to the Overall Antitumor Effect of Pegylated Liposomal Doxorubicin (Doxil^®^) in C26 Tumor-Bearing Immunocompetent Mice

**DOI:** 10.3390/pharmaceutics12100990

**Published:** 2020-10-19

**Authors:** Takuma Takayama, Taro Shimizu, Amr S. Abu Lila, Yuki Kanazawa, Hidenori Ando, Yu Ishima, Tatsuhiro Ishida

**Affiliations:** 1 Department of Pharmacokinetics and Biopharmaceutics, Institute of Biomedical Sciences, Tokushima University, 1-78-1, Sho-machi, Tokushima 770-8505, Japan; c@tokushima-u.ac.jp (T.T.); shimizu.tarou@tokushima-u.ac.jp (T.S.); c401503025@tokushima-u.ac.jp (Y.K.); h.ando@tokushima-u.ac.jp (H.A.); ishima.yuu@tokushima-u.ac.jp (Y.I.); 2 Department of Pharmaceutics and Industrial Pharmacy, Faculty of Pharmacy, Zagazig University, Zagazig 44519, Egypt; amr_selim78@yahoo.com; 3 Department of Pharmaceutics, College of Pharmacy, University of Hail, Hail 81442, Saudi Arabia

**Keywords:** antitumor immunity, chemotherapy, doxorubicin (DXR), drug delivery system, Doxil^®^

## Abstract

Doxorubicin (DXR) has been reported to have direct cytotoxicity against cancer cells and indirect immunotoxicity by modulation of host antitumor immunity. Hence, it may prevent cancer progression by a dual mechanism. Doxil^®^, a formulation of DXR encapsulated in polyethylene glycol modified (PEGylated) liposomes, is the most widely used of the clinically approved liposomal anticancer drugs. However, the effect of Doxil^®^ on host antitumor immunity is not well understood. In this study, Doxil^®^ efficiently suppressed tumor growth in immunocompetent mice bearing C26 murine colorectal carcinomas, but not in T cell-deficient nude mice, indicating a contribution of T cells to the overall antitumor effect of Doxil^®^. In immunocompetent mice, Doxil^®^ increased major histocompatibility complex (MHC-1) levels in C26 tumors, which may be an indicator of increased immunogenicity of tumor cells, and potentially amplified tumor immunogenicity by decreasing immunosuppressive cells such as regulatory T cells, tumor-associated microphages and myeloid-derived suppressor cells that collectively suppress T cell-mediated antitumor responses. This suggests that encapsulation of DXR into PEGylated liposomes increased the therapeutic efficacy of DXR though effects on host antitumor immunogenicity in addition to direct cytotoxic effects on tumor cells. This report describes the role of host antitumor immunity in the overall therapeutic effects of Doxil^®^. Manipulating pharmacokinetics and biodistribution of chemotherapeutic agents with immunomodulatory properties may increase their therapeutic efficacies by amplifying host antitumor immunity in addition to direct cytotoxic effects on tumor cells.

## 1. Introduction

Chemotherapy using cytotoxic chemicals to induce cell death, despite recent advances in alternative therapies, remains a frontline defense against malignant tumors. Besides their direct cytotoxic effects against tumor cells, many chemotherapeutic agents have been reported to destroy various immune cell populations and, consequently, impair cell-mediated innate and adaptive antitumor immunity [1]. Nevertheless, evidence is accumulating that supports the case for the contribution of host immune cells to the outcome of chemotherapy [2,3]. Certain chemotherapeutic agents could, in addition to their direct cytotoxic effect, trigger a tumor-directed immune response or increase the vulnerability of tumor cells to immune attack [4,5,6]. In this context, Aoto et al. [7] reported that treatment with chemotherapeutic agents could trigger the release of the high-mobility group box 1 protein (HMGB1), a toll-like receptor 4 (TLR4) ligand, with subsequent induction of potent tumor antigen-specific T cell immunity. In addition, Obeid et al. [8] have shown that chemotherapy-induced exposure of calreticulin, a pleiotropic molecule that has direct immunomodulatory activity, substantially enhanced the phagocytosis of dying tumor cells by dendritic cells (DCs).

Of interest, chemotherapeutic agents can also affect antitumor immunity by directly modulating the functional properties and/or survival of various immune cell populations. For example, cyclophosphamide showed the ability to deplete the frequency and/or immunosuppressive capacity of regulatory T cells (Tregs), thereby enhancing immune effector cell-mediated antitumor responses [9]. Similarly, gemcitabine, oxaliplatin and paclitaxel were reported to reduce the tumor infiltration of myeloid-derived suppressor cells (MDSCs), tumor-associated macrophages (TAMs) and/or Tregs [10,11,12,13].

Doxorubicin (DXR) is a widely used chemotherapeutic agent employed in the treatment of several types of cancers, including soft tissue sarcomas, hematological malignancies and breast cancer [14]. Accumulating evidence has established that, besides its direct antitumor activity, DXR can modulate anticancer immunity [15,16] and promote anti-tumor immunity [16,17,18].

Drug delivery systems enable the enhanced absorption, controlled release and targeting of chemotherapeutics to tumor tissues. So, many researchers have developed various types of drug delivery systems, such as nanoparticles, liposomes, micelles, emulsions and hydrogels, for the improvement of cancer chemotherapy [19,20]. Each delivery system has distinct physicochemical characteristics, including size, charge, rheology and pH. For local injections, most of the delivery systems are applicable even if they have high viscosity or non-physiological pH and osmotic pressure. However, systemic intravenous injection has some limitations for physicochemical characteristics of delivery systems [21]. Liposomes, phospholipid bilayer-based nanosized vesicles, are biocompatible and safe even after systemic intravenous injection because physicochemical characteristics of liposomes can be tuned easily [22]. Thus, liposomes have been utilized in clinical settings to improve the pharmacokinetics and tumor accumulation of encapsulated drugs.

Polyethylene glycol modified (PEGylated) liposomal anticancer drugs are employed in a number of clinical applications [23,24]. It is well known that PEGylated liposomes have, in some circumstances, the ability to preferentially accumulate interstitially in tumor tissue via a phenomenon known as the “enhanced permeability and retention (EPR) effect” [25,26]. Through changes in the pharmacokinetics and pharmacodynamics of the parent drug, PEGylated liposomal drugs had fewer and/or reduced side effects compared to conventional free drugs, and, particularly in the case of well-vascularized tumors, the encapsulated drugs had superior antitumor effects [27]. Besides any contribution of intratumor accumulation to enhancing anticancer effects, we are studying the contribution of liposome encapsulation to immunomodulatory anticancer effects [13]. Encapsulation of oxaliplatin (l-OHP) within PEGylated liposomes contributes to antitumor immunity by decreasing the number of immune suppressor cells, including TAMs, MDSCs and Tregs, which collectively suppress CD8^+^ T cell-mediated antitumor immunity [13]. On the other hand, free l-OHP suppressed antitumor immunity, presumably due to its low concentrations in the tumor tissue and its cytotoxicity against CD8^+^ T cells [13].

Doxil^®^ is a clinically approved PEGylated liposome formulation of the anticancer drug DXR-HCl, widely used in the treatment of ovarian cancer, acquired immunodeficiency syndrome (AIDS)-related Kaposi’s sarcoma and multiple myeloma [28,29], as well as a substitute for the free drug in the control of anthracycline-associated cardiomyopathies [30]. In well-vascularized tumors, the long circulation half-lives of PEGylation liposomes results in increases in interstitial tumor accumulation of DXR via the EPR effect [31], as well as some evidence that it promotes anti-tumor immunity [16,17,18]. One might assume that these two mechanisms of action of Doxil^®^, i.e., direct cytotoxic effects, combined with modulation of antitumor immunity, would increase the overall therapeutic effects, but little evidence of this exists in the literature.

In this study, we examined the contribution of host tumor immunity to the overall antitumor effects of Doxil^®^ in a C26 murine colon carcinoma model in immunocompetent or immunodeficient mice.

## 2. Materials and Methods

### 2.1. Materials

Doxil^®^ was purchased from Janssen Pharmaceutical K.K. (Tokyo, Japan). Palmitoyloleoyl phosphatidylcholine (POPC) was purchased from NOF (Tokyo, Japan). Cholesterol (CHOL) was purchased from FUJIFILM Wako Pure Chemical (Osaka, Japan). DXR-HCl (Adriamycin^®^) was purchased from Kyowa Hakko Kirin (Tokyo, Japan). Disodium clodronate tetrahydrate was purchased from Tokyo Chemical Industry Co., Ltd. (Tokyo, Japan). All other reagents were of analytical grade.

### 2.2. Animal and Tumor Cell Line

Male BALB/c and BALB/c nude mice (5–7 weeks-old, male) were purchased from Japan SLC (Shizuoka, Japan). The mice had free access to water and mouse chow and were housed under controlled environmental conditions (constant temperature, humidity and a 12 h dark/light cycle). All animal experiments were evaluated and approved by the Animal and Ethics Review Committee of Tokushima University.

Colon 26 (C26) murine colorectal carcinoma cells were obtained from the Cell Resource Center for Biomedical Research (RIKEN RBC CELL BANK, Saitama, Japan). The C26 cell line was maintained in Dulbecco’s Modified Eagle Medium (DMEM), supplemented with 10% heat-inactivated fetal bovine serum (FBS, Sigma, St. Louis, MO, USA), with 100 units/mL penicillin and 100 μg/mL streptomycin (FUJIFILM Wako Pure Chemical) in a 5% CO_2_/air incubator at 37 °C.

### 2.3. Preparation of Clodronate-Containing Liposomes

Clodronate-containing liposomes, composed of POPC/CHOL (45/8, molar ratio), were prepared by a thin film evaporation technique, as described previously [32,33]. Briefly, the lipids were dissolved in chloroform, and the organic solvent was removed by rotary evaporator under vacuum at 37 °C. The lipid film was then hydrated with 10 mL of phosphate-buffered saline (PBS) containing 2.5 g of disodium clodronate tetrahydrate. The liposomes were incubated under nitrogen for 2 h, sonicated for 3 min and then incubated under nitrogen for another 2 h. The liposomes were frozen at −80 °C for 2 h and thawed at 37 °C for 15 min. The liposomes were sized by sequential extrusion through polycarbonate membrane filters (Whatman Inc., Florham Park, NJ, USA) with pore sizes of 400, 200 and 100 nm. Free clodronate was separated from clodronate liposomes by centrifugation (30 min at 100,000× *g*). The pellets were re-suspended in PBS. The final lipid concentration was 33 µmol/mL. Particle size of the prepared liposomes was determined using a Zetasizer Nano ZS (Malvern Instruments, Worcestershire, UK) and was 114 ± 41.2 nm. The resulting liposomes contained approximately 5 mg/mL clodronate.

### 2.4. Antitumor Effect of DXR Formulations

C26 cells (2 × 10^6^) were inoculated subcutaneously (s.c.) in the left flank of either BALB/c or BALB/c nude mice. The date when tumor volume reached 100–150 mm^3^ was set at Day 0. The mice were randomized into three groups (*n* = 5 in each group). The first group, receiving PBS on Day 0 and Day 5, served as a control group. The second group was injected intravenously (i.v.) with free DXR (5 mg/kg) on Day 0 and Day 5. The third group was injected i.v. with Doxil (5 mg DXR/kg) on Day 0 and Day 5.

To see the effect of macrophages on antitumor activity of DXR formulations, clodronate-containing liposomes were injected (6.6 µmol phospholipids/mouse) i.v. together with either DXR or Doxil (5 mg DXR/kg) on Day 0 and Day 5.

Estimation of tumor volume was conducted using a caliper, and tumor volume was calculated using the following formula [34]:

Tumor volume (mm^3^) = (width)^2^ × (length)/2

Body weight was measured at the same time, as an indicator for systemic toxicity.

### 2.5. Effect of Treatment with DXR Formulations on Immune Cell Populations in the Tumor Tissue

Mice were injected i.v. with either free DXR (5 mg/kg) or Doxil^®^ (5 mg DXR/kg) on Day 0 and Day 5. Tumors were harvested on day 11 and tumor tissues were digested with collagenase type I (FUJIFILM Wako Pure Chemical) and Dispase II (Roche Diagnostic, Mannheim, Germany). Cell suspensions were prepared using a gentle MACS octo dissociator (MiltenyiBiotec, Bergisch Gladbach, Germany), as described previously [13]. To stain immune populations, cells were incubated with the combinations of antibodies as follows: CD8^+^ T cells, anti-mouse CD8a-eFluor 660; MDSC, anti-mouse Ly-6G (Gr-1)-eFluor 660 and anti-mouse CD11b-fluorescein isothiocyanate (FITC); Treg, anti-mouse CD4-eFuor 660 and anti-mouse CD25-Alexa Fluor 488; TAM, anti-mouse F4/80-eFluor 660, (eBioscience, San Diego, CA, USA) and anti-mouse CD206 (MMR)-Alexa Fluor 488 (BioLegend, San Diego, CA, USA)) for 30 min at 4 °C. Fluorescence data were collected on a flow cytometer (Gallios, Beckman Coulter, Brea, CA, USA). The data were analyzed using Kaluza 1.2 software (Beckman Coulter).

### 2.6. Evaluation of MHC Class I Expression on Tumor Cells

C26 cells (1 × 10^5^ cells) were seeded onto 12-well plates. After 24 h incubation, the culture medium was replaced with fresh media containing either free DXR or Doxil^®^ (1, 5, 20, 40 DXR µM). After a further 24 h incubation, cells were harvested and then incubated with the anti-mouse MHC class 1 (H-2Dd) antibody (AbDserotec, Oxford, UK) for 30 min at 4 °C. After two washes, the cells were further stained with the indicated antibodies (goat anti-mouse lgG-Alexa Fluor 647, Thermo Fisher Scientific, Waltham, MA, USA) for 30 min at 4 °C. Fluorescence data were analyzed by flow cytometry as described above.

To evaluate MHC class I expression on tumor cells in vivo, mice were injected i.v. with either free DXR or Doxil^®^ (5 mg DXR/kg) on Day 0 and Day 5. Tumor was harvested on Day 11 and cell suspensions were prepared as described above. MHC class I expression level was analyzed by flow cytometry as mentioned above.

### 2.7. Statistical Analysis

All values are presented as mean ± standard deviation (SD). Statistical analysis was performed by one-way analysis of variance (ANOVA) followed by Tukey’s post hoc testing using GraphPadInStat software (GraphPad Software, La Jolla, CA, USA). The level of significance was set at *p* < 0.05.

## 3. Results

### 3.1. Tumor Growth Suppressive Effects of DXR Formulations in C26 Tumor-Bearing Immunocompetent Versus Immunodeficient Nude Mouse Models

**Figure 1:**
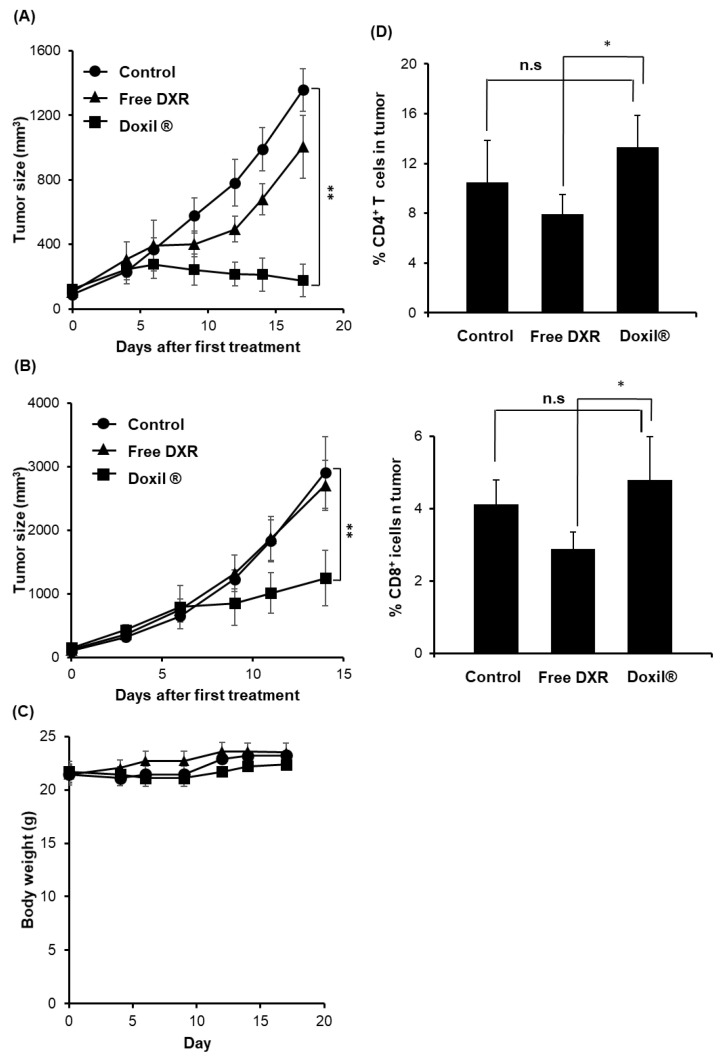
Antitumor effects of Doxil^®^ and free doxorubicin (DXR) in C26 tumor-bearing immunocompetent or immunodeficient mice. Either BALB/c mice or BALB/c nude mice were inoculated s.c. with C26 cells (2 × 10^6^ cells). The day when tumor volumes reached 100–150 mm^3^ was set as Day 0. The mice were randomized into three groups (n = 5 in each group). On Day 0 and Day 5, the mice were injected i.v. with phosphate buffer saline (PBS, control), free DXR (5 mg/kg) or Doxil^®^ (5 mg DXR/kg). (**A**) Tumor growth in immunocompetent mice, (**B**) tumor growth in immunodeficient mice. Each value represents the mean ± SD. ** *p* < 0.01. (**C**) Body weight change in immunocompetent mice. (**D**) The ratio of CD4^+^ T cells and CD8^+^ T cells to total cells in the treated tumors were analyzed by flow cytometry. Each value represents the mean ± SD. * *p* < 0.05, ** *p* < 0.01.

Either immunocompetent or immunodeficient (lacking T cells) mice, bearing C26 tumors, were treated with two i.v. injections of PBS, free DXR (5 mg/kg) or Doxil^®^ (5 mg DXR/kg). In the tumor-bearing immunocompetent mice, treatment with free DXR resulted in marginal, non-significant reductions in tumor size (*p* > 0.05), compared to the PBS-treated control group (Figure 1A). Treatment with Doxil^®^ significantly suppressed tumor growth compared to the other groups (*p* < 0.01), which is consistent with previous studies [27,35]. Interestingly, in immunodeficient mice, tumor growth rates were more rapid than those in immunocompetent mice. In the tumor-bearing immunodeficient nude mice, free DXR failed to exert any tumor growth suppression, compared to the PBS-treated control group (Figure 1B). Doxil^®^ induced significant tumor growth suppression (*p* < 0.01) as observed in Figure 1B. However, the antitumor effect of Doxil^®^ observed in the immunocompetent mice was higher than that in immunodeficient mice. The tumor growth inhibition rate was 0.57 in tumor-bearing immunodeficient mice and 0.87 in tumor-bearing immunocompetent mice, respectively. Throughout these therapeutic experiments with immunocompetent and immunodeficient mice, no obvious weight losses relating to severe side effects were observed (Figure 1C). These results suggest that immune cells such as T cells influence tumor growth in mice and contribute to the overall therapeutic effect of Doxil^®^.

To gain more insight into the possible contribution of T cell-mediated antitumor immunity to Doxil^®^-mediated tumor growth suppression in the immunocompetent mice, levels of both CD8^+^ and CD4^+^ T cells in tumor tissue of immunocompetent mice was determined following treatment with either free DXR or Doxil^®^ (Figure 1D, Supplementary Figure S1). Treatment with free DXR tended to decrease the number of both tumor infiltrating CD8^+^ and CD4^+^ T cells (non-significant, n.s.), which might be due to non-specific toxicity. On the other hand, Doxil^®^ treatment, if anything, tended to increase levels (n.s.) of both tumor infiltrating CD8^+^ and CD4^+^ T cells (Figure 1D, Supplementary Figure S1). These results suggest that T cell-mediated antitumor immunity may make some contribution to the overall therapeutic efficacy of Doxil^®^ in immunocompetent mice.

### 3.2. Effect of Treatment with Doxil^®^ on Protumor Host Immunity

**Figure 2:**
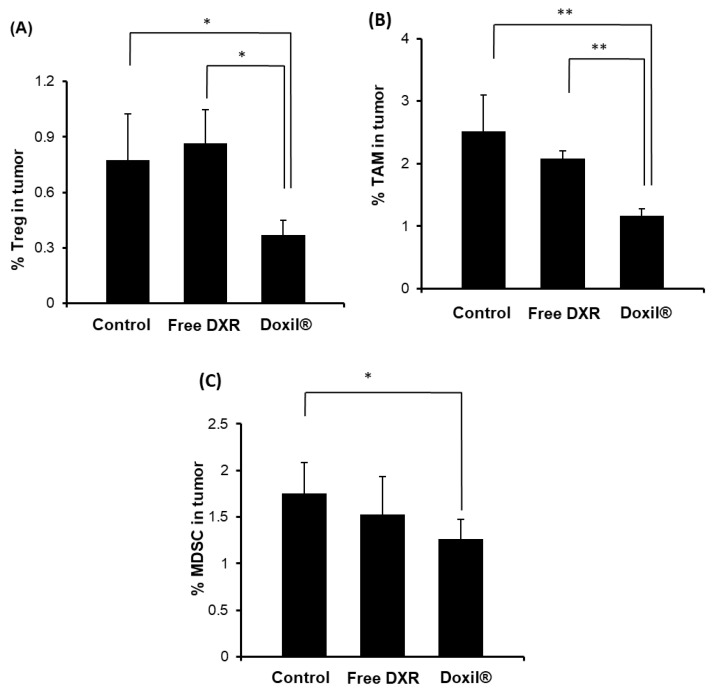
Effect of treatment with either free DXR or Doxil^®^ on the numbers of immunosuppressive cells in the tumor of immunocompetent mice. BALB/c mice were inoculated s.c. with C26 cells (2 × 10^6^ cells). The day when tumor volumes reached 100–150 mm^3^ was set as Day 0. Mice were injected i.v. with PBS, free DXR (5 mg/kg) or Doxil^®^ (5 mg DXR/kg) on Day 0 and Day 5. Tumor was harvested on Day 11. The ratio of (**A**) regulatory T cell (Treg), (**B**) tumor-associated macrophage (TAM), (**C**) myeloid-derived suppressor cell (MDSC) to total tumor cells were analyzed by flow cytometry. Each value represents the mean ± SD. * *p* < 0.05, ** *p* < 0.01.

It is well known that there is a balance between pro- and anti-tumor immunity [36,37]. Accordingly, we investigated the effect of treatment with either free DXR or Doxil^®^ on the immunosuppressive cell populations in the tumor tissues of immunocompetent mice. Doxil^®^, but not free DXR, significantly reduced the numbers of Tregs (Figure 2A, Supplementary Figure S1). Doxil^®^ significantly reduced the number of TAMs (Figure 2B, Supplementary Figure S1), but free DXR tended to decrease (n.s.) their numbers. In addition, the treatment with Doxil significantly skewed polarization from M2 phenotype to the M1 phenotype, which contributes to antitumor immunity (Supplementary Figure S2). Likewise, Doxil^®^ somewhat reduced the numbers of MDSCs (*p* < 0.05), but free DXR did not (Figure 2C, Supplementary Figure S1). These results indicate that the treatment with Doxil^®^ can somewhat reduce the numbers of immunosuppressive cells, in particular Tregs and TAMs, in tumor tissues in immunocompetent mice.

### 3.3. Effect of Depletion of TAMs in the Tumor Tissues on Doxil^®^-Mediated Tumor Growth Suppression in Immunocompetent Mice

Protumor immune cells such as MDSCs and TAMs have phagocytic activity, but Tregs do not [38]. Liposomes are known to be taken up by phagocytic cells [39]. In our preliminarily experiments, we confirmed a higher uptake of Doxil^®^ by TAMs, and to a lesser extent, by MDSCs in the tumor tissues (Supplementary Figure S3). This might explain the somewhat reduced number of TAMs and MDSCs in tumor tissue following treatment with Doxil^®^ (Figure 2B). Published reports have emphasized an important role of TAM in suppression of antitumor T cell responses via secretion of interleukin 10 (IL-10) and transforming growth factor beta (TGF-β) [37]. Also, liposomes encapsulating alendronate, a bisphosphonate drug that can decrease white blood cell levels, are reported to suppress tumor growth via depletion of TAM. One can speculate that the elimination of protumor immune cells such as MDSCs and TAMs from tumor tissues by Doxil^®^ might play a role in increasing tumor-specific immune responses, working together with DXR cytotoxicity. Therefore, antitumor effects were evaluated for free DXR in immunocompetent mice depleted of phagocytic cells

**Figure 3:**
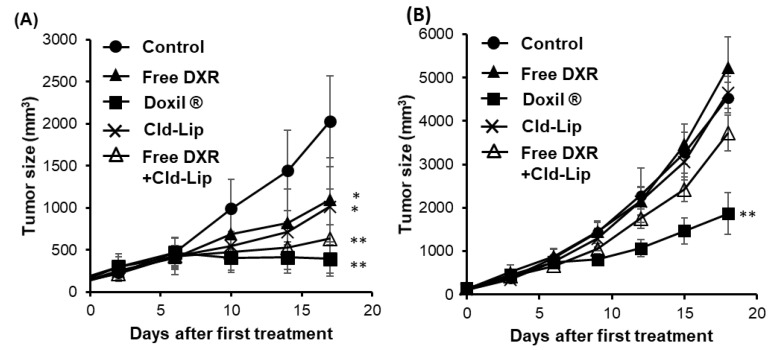
Effect of depletion of phagocytic cells, particularly TAMs, on antitumor effects of Doxil^®^ and free DXR in C26 tumor-bearing immunocompetent or immunodeficient mice. Mice were inoculated s.c. with C26 cells (2 × 10^6^ cells). The day when tumor volumes reached 100–150 mm^3^ was set as Day 0. The mice were randomized into five groups (*n* = 5 in each group). On Day 0 and Day 5, mice were injected i.v. with PBS, free DXR (5 mg/kg) or Doxil^®^ (5 mg DXR/kg), clodronate liposome (Cld-Lip, 6.6 µmol/mouse) or a combination of free DXR (5 mg/kg) plus Cld-Lip (6.6 µmol/mouse). (**A**) Tumor growth in immunocompetent mice. (**B**) Tumor growth in immunodeficient, T cell-deficient, mice. Each value represents the mean ± SD. * *p* < 0.05, ** *p* < 0.01.

Depletion of phagocytic cells, including TAMs, was achieved by i.v. administration of the macrophage inhibitor, clodronate liposomes [40] (Supplementary Figure S4). In C26-bearing immunocompetent mice, treatment with clodronate liposomes caused some increase in tumor growth suppression (*p* < 0.05), of a similar level to that of free DXR treatment (Figure 3A). Concomitant administration of free DXR with clodronate liposome additively increased the tumor growth suppressive effect of free DXR *(p <* 0.01), which was close to the levels of tumor suppression with Doxil^®^ treatment. In T cell-deficient nude mice, Doxil^®^ showed increased tumor growth suppression, relative to free DXR, clodronate liposomes or the combination (Figure 3B). These results suggest that the phagocytic cells in the tumor tissues, mainly TAMs, which are eliminated by clodronate liposomes, can contribute to the antitumor potency of Doxil^®^, amplifying to a small extent cytotoxicity of free DXR in this particular tumor model.

### 3.4. Changes Caused by either Doxil^®^ or Free DXR Treatment on MHC I Expression Levels on Tumor Cells

**Figure 4:**
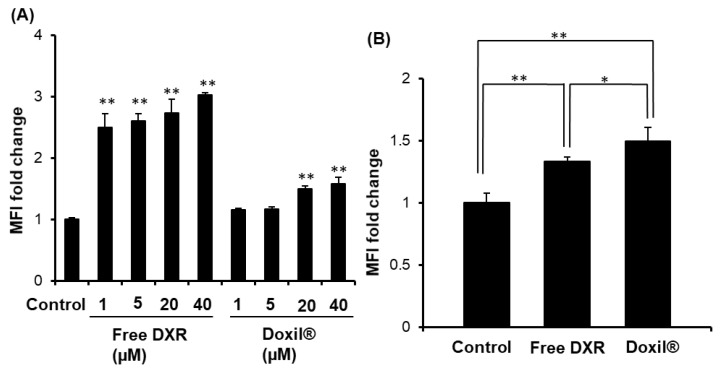
Change of major histocompatibility complex (MHC I) expression levels on tumor cells by either Doxil^®^ or free DXR treatment. (**A**) C26 cells (1 × 10^5^ cells/well) were cultured in a 12-well plate for 24 h in the presence of various concentrations of either free DXR or Doxil^®^ (1, 5, 20, 40 µM). Cells were then harvested in EDTA-PBS. MHC class I presentation levels on the cells were analyzed by flow cytometry. (**B**) Immunocompetent mice were inoculated s.c. with C26 cells (2 × 10^6^ cells). The day when tumor volumes reached 100–150 mm^3^ was set as Day 0. On day 0 and Day 5, C26 tumor-bearing mice were injected i.v. with either free DXR (5 mg/kg) or Doxil^®^ (5 mg DXR/kg). On Day 11, tumors were harvested. MHC class I presentation levels on the cells were analyzed by flow cytometry. Each value represents the mean ± SD. * *p* < 0.05, ** *p* < 0.01.

MHC class I downregulation is a well-recognized mechanism used by tumor cells to evade destruction by antitumor cytotoxic T lymphocytes [41]. The effect was studied of treatment with either free DXR or Doxil^®^ on the level of MHC-1 in C26 tumor cells in vitro and in vivo. Under in vitro conditions, treatment with free DXR increased MHC class I-expressions on C26 tumor cells at all DXR concentrations we tested (Figure 4A, Supplementary Figure S1). Treatment with Doxil^®^ also increased the expressions of MHC-1, but to a lesser extent and only at the two highest DXR concentrations. Under in vivo conditions, either free DXR or Doxil^®^ increased the expression levels of MHC class 1 on the tumor cells to a small degree (Figure 4B, Supplementary Figure S1), with Doxil^®^-treated tumor cells showing somewhat higher levels of MHC class I expression than free DXR-treated tumor cells (*p* < 0.05). Hence, Doxil^®^ appears to increase the MHC class 1 levels on the tumor cells in vivo, corresponding to increased tumor cell antigenicity.

## 4. Discussion

Conventional anticancer drugs are broadly cytotoxic and are generally thought to exert their tumor growth suppressive effects via direct killing of tumor cells and immunosuppression via severe myelosuppression. Nevertheless, accumulating evidence has highlighted the contribution of the host immune system to the overall antitumor efficacy of several chemotherapeutic agents [2,3,13,42]. In the current study, Doxil^®^ treatment resulted in higher antitumor effects in tumor-bearing immunocompetent mice than in tumor-bearing immunodeficient nude mice (Figure 1). This suggests that T cells played a role in the antitumor effects of Doxil^®^. Our current study emphasizes the contribution of host antitumor immunity to the overall therapeutic efficacy of Doxil^®^.

The development of malignant tumors is commonly associated with the occurrence and persistence of an immunosuppressive environment [15]. In the current study, Doxil^®^ reduced the number of Tregs, TAMs and MDSCs from tumor tissue of immunocompetent mice (Figure 2) and Doxil^®^ skewed polarization from M2 to M1 phenotype (Supplementary Figure S2), which would lead to an increase in CD4^+^/CD8^+^ T cell-mediated antitumor immunity. In addition, Doxil^®^ upregulated levels of MHC class 1 expression in the tumors of immunocompetent mice (Figure 4B), rendering them vulnerable to CD8^+^ T cells in vivo. In immunocompetent mice, this would tend to reduce tumor-induced host antitumor immunity suppression and add to the direct anti-tumor cytotoxic effects of Doxil^®^ (Figure 1A). Despite its well-established tumor cytotoxicity and its immunosuppressive effect [43,44], free DXR failed to exert a substantial tumor growth suppressive effects, compared to Doxil^®^, in tumor-bearing immunocompetent mice under our experimental conditions (Figure 1A). This might result from only a modest reduction in the number of CD4^+^/CD8^+^ T cells (Figure 1D) and no/little reduction in the number of Tregs, TAMs and MDSCs (Figure 2) in vivo, due to lower intratumor DXR concentrations compared to Doxil^®^ [31].

It is well known that Doxil^®^ preferentially accumulates in solid tumors via the EPR effect, in well-vascularized tumors, and achieves elevated intratumoral DXR concentrations [31]. Besides promoting intratumor accumulation of the encapsulated payload, liposome entrapment of DXR may have other advantages, e.g., in helping to promote antitumor immunity, since liposomes are taken up by phagocytic cells. In the current study, uptake experiments demonstrated a higher propensity for uptake of Doxil^®^ by protumor immune cells such as TAMs and MDSCs (Supplementary Figure S3), which have phagocytic activity. This would result in a reduction in the numbers of TAMs (Figure 2B) and MDSCs (Figure 2C) in the tumor tissue of immunocompetent mice. Consequently, the ratios of CD4^+^ and CD8^+^ T cells to MDSCs or TAMs were increased following Doxil^®^ treatment, which is anticipated to contribute, at least in part, to the increased antitumor efficacy of Doxil^®^ in the tumor-bearing mice compared to the free drug (Figure 1A). In addition to its preferential tumor accumulation, the sustained drug release properties of Doxil^®^ mimics infusion of free drug, resulting in exposure of tumor cells to drug for longer periods of time. This might contribute to reductions in the numbers of Tregs (Figure 2A), which do not have phagocytic activity, and increase the MHC-1 levels in tumors, promoting immunogenic death, contributing to the overall tumor growth suppressive effect of Doxil^®^.

Macrophages are among the most abundant cells in the tumor microenvironment and can account for up to 50% of tumor mass [45]. Extensive evidence emphasized the crucial role of TAMs in tumor progression and metastasis [46,47,48]. In addition, several studies have investigated the efficacy of chemotherapeutic agents in subverting the pro-tumorigenic activities of TAMs [49,50,51]. In this study, depletion of phagocytic cells, including TAM, from tumor tissue by clodronate liposomes (Supplementary Figure S4) suppressed tumor growth in immunocompetent mice (Figure 3A), which is consistent with the reports of the other groups [40,52]. The combined treatment with free DXR plus clodronate liposomes suppressed tumor growth in the immunocompetent mice, at a level comparable to that seen for Doxil^®^ treatment (Figure 3A). These results support a role for the suppression of protumor immune cells including TAMs in the antitumor effects of Doxil^®^ in the immunocompetent mice.

Our previous studies, as well as the current study, show that liposomal oxaliplatin and liposomal clodronate decreased in vivo levels of protumor immune cells, including TAM, but failed to suppress the tumor growth in T cell-deficient mice. Although Doxil^®^ significantly suppressed tumor growth in the absence of T cells, probably due to direct cytotoxicity against tumor cells, the tumor suppressing effect was decreased by a lack of T cells. These observations support previous data demonstrating the indispensable role of T cells in tumor growth suppression, even when protumor immunity was decreased by liposomal chemotherapeutics. However, in preclinical studies, athymic nude mice (lacking T cells) and severe combined immunodeficiency (SCID) mice (lacking T cells and B cells) were used as tumor xenograft models because of the difficulty of inoculation of human cancer cells into immunocompetent mice. Under such conditions, the efficacy of liposomal chemotherapeutics might be underestimated in cases when they have immunomodulatory effects. In addition, therapeutic indications for liposomal drugs have been proposed based on their direct anti-tumor cytotoxic effects, not on any immunomodulatory effects that might be occurring against the tumor immune microenvironment, which contributes to the resistance to cancer cells against treatment. Therefore, selection of chemotherapeutic agents with immunomodulatory properties might identify promising candidates that could increase therapeutic outcomes, especially against resistant tumors.

## 5. Conclusions

We demonstrated that, besides its direct cytotoxic effect of entrapped free DXR, Doxil^®^ could indirectly increase antitumor immune effects by eliminating immunosuppressive cells. Doxil^®^ upregulated MHC class 1 expression level on C-26 tumor cells, leading to enhanced tumor cell susceptibility to T cell-mediated cytotoxicity. Liposome formulations of chemotherapeutic agents, having immunomodulatory properties, may, with appropriate attention to selection criteria, lead to increased therapeutic efficacy by increasing host antitumor immunity in support of their direct cytotoxic effects on tumor cells.

## Supplementary Materials

The following are available online at https://www.mdpi.com/1999-4923/12/10/990/s1, Figure S1: Gating strategies and representative plot of flow cytometry analysis. Figure S2: Polarization of Tam in tumor tissues after treatment with Doxil. Figure S3: Cellular uptake of Doxil^®^ by immune cells in tumor tissue. Figure S4: Effect of treatment with clodronate liposomes on TAMs in tumor tissues.
