# Supplementary material for: Adjuvant Antitumor Immunity Contributes to the Overall Antitumor Effect of Pegylated Liposomal Doxorubicin (Doxil®) in C26 Tumor-Bearing Immunocompetent Mice
Source: Pharmaceutics. 2020 Oct 19;12(10):990. doi: 10.3390/pharmaceutics12100990 (PMC7589973; doi:10.3390/pharmaceutics12100990)
Supplement: Supplementary file 1 [file pharmaceutics-12-00990-s001.zip › pharmaceutics-942759-supplementary.docx]

Supplementary Materials: Adjuvant Antitumor Immunity Contributes to the Overall Antitumor Effect of Pegylated Liposomal Doxorubicin (Doxil^®^) In C26 Tumor-Bearing Immunocompetent Mice

Takuma Takayama ^1^, Taro Shimizu ^1^, Amr S. Abu Lila ^1,2,3^, Yuki Kanazawa ^1^, Hidenori Ando ^1^,
Yu Ishima ^1^ and Tatsuhiro Ishida ^1,^*


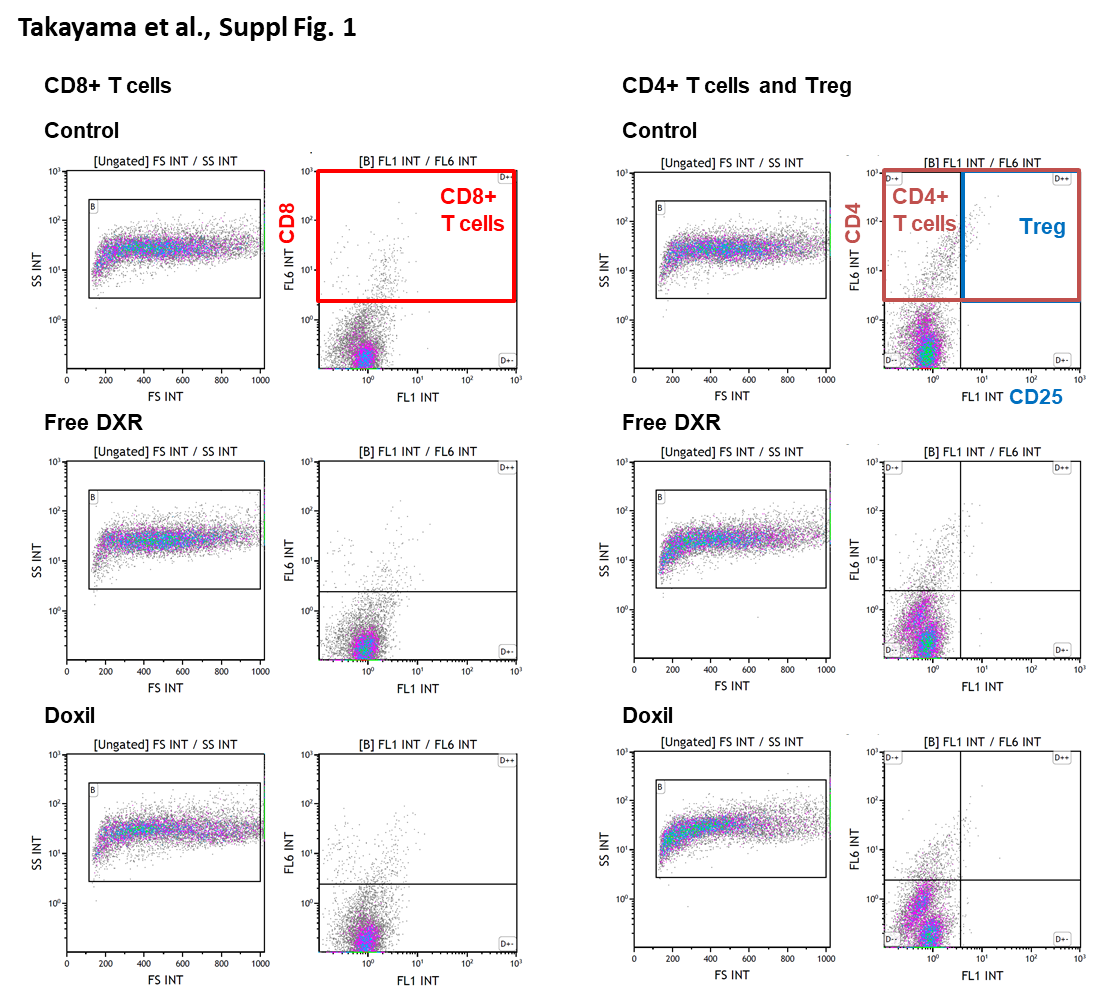


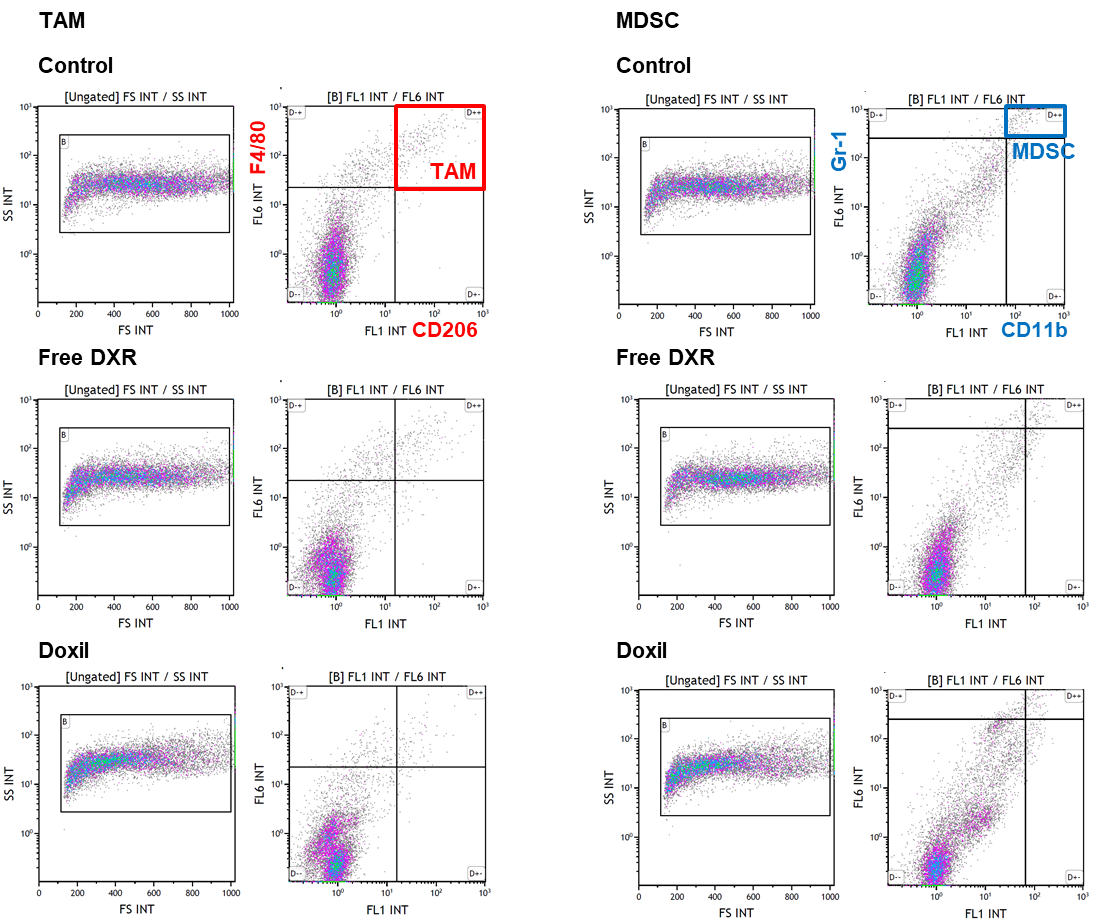


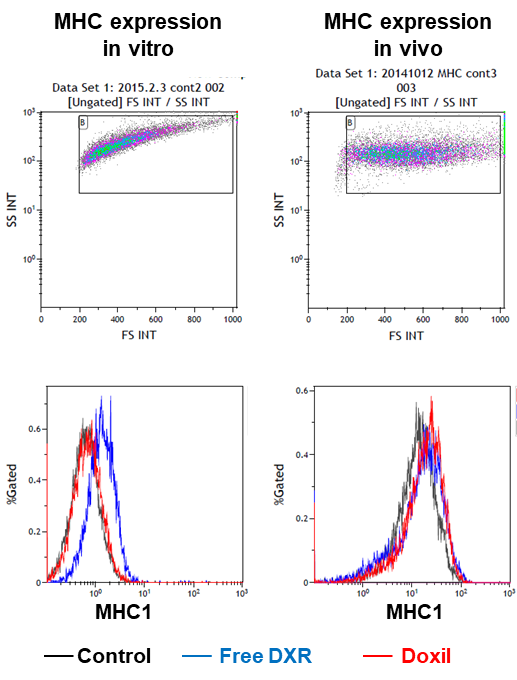


**Figure S1.** Gating strategies and representative plot of flow cytometry analysis.


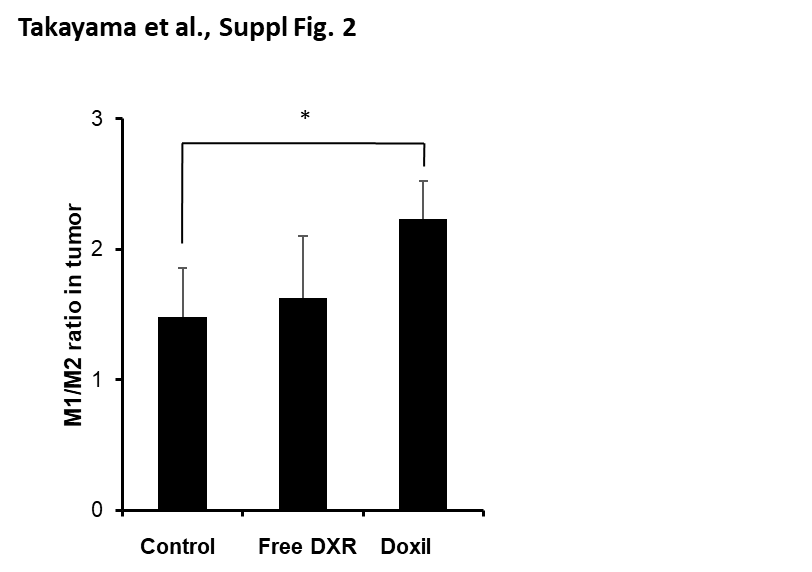


**Figure S2.** Polarization of Tam in tumor tissues after treatment with Doxil. BALB/c mice were inoculated s.c. with C26 cells (2 × 10^6^ cells). The day when tumor volumes reached 100–150 mm^3^ was set at Day 0. Mice were injected i.v. with PBS, free DXR (5 mg/kg) or Doxil^®^ (5 mg DXR/kg) on Day 0 and Day 5. Tumor was harvested on Day 11. The ratio of M1 macrophages (CD206-, F4/80+ cells) to M2 macrophages (CD206+, F4/80+ cells) were analyzed by flow cytometry. Each value represents the mean ± SD. * *p* < 0.05.


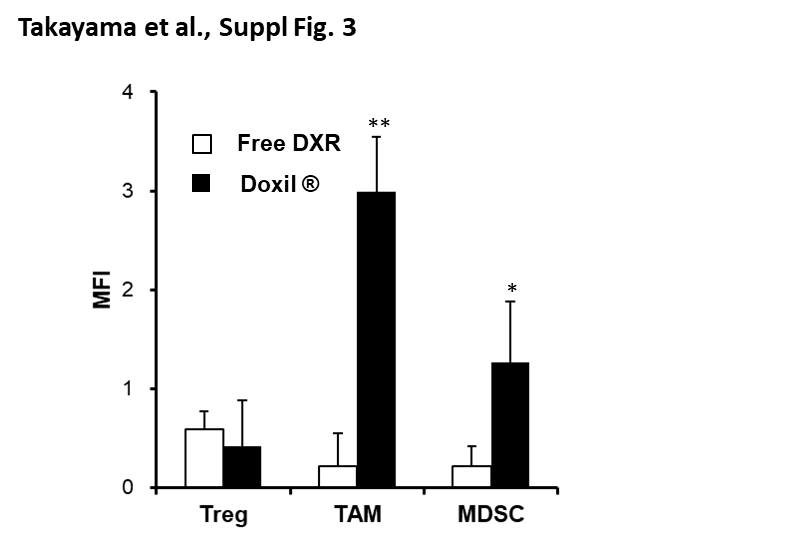


**Figure S3.** Cellular uptake of Doxil^®^ by immune cells in tumor tissue. BALB/c mice were inoculated s.c. with C26 cells (2 × 10^6^ cells). The day when tumor volume reached 100–150 mm^3^ was set at Day 0. Mice were injected i.v. with either free DXR or Doxil^®^ (10 mg DXR/kg) on Day 0. Tumor was harvested on Day 2. Fluorescence derived from DXR in Treg, TAM or MDSC was detected by flow cytometry. Each value represents the mean ± SD. * *p* < 0.05, ** *p* < 0.01 vs. DXR.


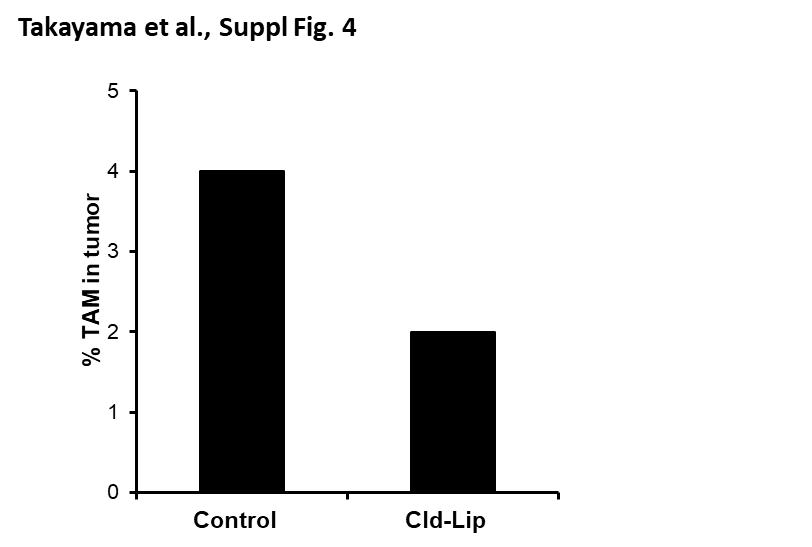


**Figure S4.** Effect of treatment with clodronate liposomes on TAMs in tumor tissues. C26 tumor-bearing immunocompetent mice were injected i.v. with Cld-Lip (6.6 µmol/mouse). Tumors were excised on Day 4. The proportion of TAMs in total tumor cells of tumor was analyzed by flow cytometry. Each value represents the mean ± SD.
